# Polyaniline as a Nitrogen Source and Lignosulfonate as a Sulphur Source for the Preparation of the Porous Carbon Adsorption of Dyes and Heavy Metal Ions

**DOI:** 10.3390/polym15234515

**Published:** 2023-11-24

**Authors:** Wenjuan Wu, Penghui Li, Wanting Su, Zifei Yan, Xinyan Wang, Siyu Xu, Yumeng Wei, Caiwen Wu

**Affiliations:** 1 Jiangsu Co-Innovation Center of Efficient Processing and Utilization of Forest Resources, Nanjing Forestry University, Nanjing 210037, China; liph@njfu.edu.cn (P.L.); xsy121517@njfu.edu.cn (S.X.); caiwenwu@njfu.edu.cn (C.W.); 2 College of Light Industry and Food Engineering, Nanjing Forestry University, Nanjing 210037, Chinayan_zifei@163.com (Z.Y.); wangxinyan0813@163.com (X.W.); yyqx@njfu.edu.cn (Y.W.)

**Keywords:** lignin, PANI, carbon, adsorption, dye

## Abstract

Using agricultural and forestry wastes as raw materials, adsorbent materials were prepared for dye adsorption in wastewater, which can minimize the environmental load and fully realize sustainability by treating waste with waste. Taking lignosulfonate as a raw material, due to its molecular structure having more reactive groups, it is easy to form composite materials via a chemical oxidation reaction with an aniline monomer. After that, using a sodium lignosulfonate/polyaniline composite as the precursor, the activated high-temperature pyrolysis process is used to prepare porous carbon materials with controllable morphology, structure, oxygen, sulfur, and nitrogen content, which opens up a new way for the preparation of functional carbon materials. When the prepared O-N-S co-doped activated carbon materials (SNC) were used as adsorbents, the adsorption study of cationic dye methylene blue was carried out, and the removal rate of SNC could reach up to 99.53% in a methylene blue solution with an initial concentration of 100 mg/L, which was much higher than that of undoped lignocellulosic carbon materials, and the kinetic model conformed to the pseudo-second-order kinetic model. The adsorption equilibrium amount of NC (lignosulfonate-free) and SNC reached 478.30 mg/g and 509.00 mg/g, respectively, at an initial concentration of 500 mg/L, which was consistent with the Langmuir adsorption isothermal model, and the adsorption of methylene blue on the surface of the carbon material was a monomolecular layer. The adsorption of methylene blue dye on the carbon-based adsorbent was confirmed to be a spontaneous and feasible adsorption process by thermodynamic parameters. Finally, the adsorption of SNC on methylene blue, rhodamine B, Congo red, and methyl orange dyes were compared, and it was found that the material adsorbed cationic dyes better. Furthermore, we also studied the adsorption of SNC on different kinds of heavy metal ions and found that its adsorption selectivity is better for Cr^3+^ and Pb^2+^ ions.

## 1. Introduction

The scarcity of forest resources has become a global environmental and energy problem. Forests are important ecosystems on Earth, maintaining the global ecological balance and providing a large amount of raw materials for human life [1]. With the increase in population, part of the forest land is transformed into agricultural land and other types of land, and a large number of trees are cut down, resulting in the reduction of forest resources. The total area of global forests was 4128 million hectares in 1990, and it was reduced to 3999 hectares in 2015 [2]. The development of inexhaustible biomass resources and bio-based new materials has become an important way to solve the forest resources crisis faced by modern society [3]. Lignocellulose is an extremely rich renewable resource on Earth, and its total carbon accounts for about 30% of the global organic carbon [4]. The main components of lignocellulosic fibers are cellulose, hemicellulose, and lignin, of which lignin has the most complex structure and is an amorphous, cross-linked three-dimensional network macromolecule [5]. Lignin is rich in renewable natural aromatic biopolymers, and it has many functional groups in its structure, such as phenolic hydroxyl, alcoholic hydroxyl, carbonyl, and methoxyl, so it can not only be used as an alternative raw material for aromatic chemical products but also has a great potential for development in many fields [6,7]. Porous carbon materials with a large specific surface area, highly developed pore structures, and good thermal stability are easy to mass-produce and widely used in energy storage, adsorption, and other fields [8,9]. Activated carbon synthesized from natural carbon sources such as biomass, biomass waste, and industrial waste has been a hot research topic in recent years due to the high availability of raw materials [10]. The paper industry produces a large amount of industrial waste lignosulfonate every year. With up to 60% carbon, lignin also has high aromaticity, high calorific value, and abundant active sites, which makes it an ideal carbon source for constructing activated carbon [8]. Qu et al. [11] reported a method to transform lignin into pore-rich foamy carbon via heating only, and the resulting lignin-derived carbon has a bulk density of up to 0.62 g·cm^−3^ with excellent strength properties. Silva et al. [12] selected lignin residual from the second-generation ethanol process as a raw material and prepared it into activated carbon using the chemical activation method. This lignin-based activated carbon is the only activated carbon that can remove bromocresol green and also has excellent adsorption performance for methylene blue (MB) and methyl red dyes, with removal rates of 96.8% and 94.1%, respectively. Polyaniline (PANI) is a polymer made through the reaction of aniline in aqueous hydrochloric acid and ammonium persulfate. It is simple to synthesize, environmentally friendly, and also possesses unique redox behavior, significant electrical conductivity, corrosion resistance, high-temperature resistance, and easy to blend with other materials [13]. The structural composition of PANI’s electronic group, amino group, and its imide derivatives provide it with significant chelating properties. In addition, its high specific surface area and porosity, the coexistence of benzoquinone ring, hole conducting nature, and positive charge acquired during protonation are important factors for its excellent dye adsorption capacity [14]. Nerka et al. [15] prepared PANI/ZnO nanocomposites using in situ polymerization for the removal of methyl orange dye. The experimental results showed improved removal of methyl orange from this composite as compared to pure ZnO nanoparticles and pure PANI.

Reactive dyes are often used in various industrial production, such as clothing [16], paper printing [17], leather [18], and cosmetics [19]. With the rapid development of industrial technology, there has been an exponential growth of polluted wastewater containing large amounts of residual dyes and dyeing aids [20]. Dyes are severely toxic, carcinogenic, and adversely affect human and animal health when entering water bodies, and their complex molecular structure makes them difficult to degrade [21]. Heavy metal pollution is also one of the important factors leading to pollution and shortage of water bodies. These heavy metal ions present in water are difficult to convert into non-toxic and non-hazardous substances using biodegradation as compared to organic pollutants [22]. Heavy metals in water can be easily absorbed by living organisms and then enter the food chain and accumulate in the organisms, thus threatening the life and health of the organisms [23]. Therefore, how to manage industrial wastewater containing reactive dyes and metal ions has become an important proposition facing the world. Carbon materials, with a high specific surface area and special surface chemistry, show excellent pollutant adsorption capacity in wastewater treatment [24]. Compared to ordinary carbon materials, carbon materials doped with heteroatoms increase the chemisorption of carbon materials due to the increased surface defects and chemically active sites of the material [25]. Heteroatom-doped carbon materials have better adsorption properties and can be well used for the adsorption and separation of various pollutants. Based on this, Shi et al. [26] proposed to prepare a novel Cu/N doped modified lignin adsorbent by reacting lignin with triethylenetetramine and CuCl_2_ as raw materials, and the adsorption amount of As(V) was 253.5 mg/g. This suggests that the pollutant adsorption capacity of the lignin carbon material can be effectively enhanced by doping with the heteroatoms N. In this work, nanostructured lignin/polyaniline complexes were successfully prepared from lignin using a simple and controllable chemical oxidative polymerization method and used as prepolymers and porous oxygen/nitrogen/sulfur co-doped activated carbon materials were prepared via KOH activation and carbonization. The effects of the preparation conditions on the morphology, structure, and properties of the products were explored, and the adsorption properties of dyes from lignin/polyaniline porous carbon materials were investigated. In particular, we also used SNC to test the adsorption capacity of different dye molecules for different heavy metal ions.

## 2. Materials and Methods

### 2.1. Materials

Lignosulfonate, methylene blue, rhodamine B (RhB), Congo red (CR), methyl orange (MO), and Cr^3+^, Pb^2+^, Ni^2+^, Cu^2+^, Zn^2+^, and Cd^2+^ standard solutions were from Aladdin Reagent Co. (Shanghai, China). Aniline and ammonium persulfate were purchased from Macklin Reagent Co. (Shanghai, China). KOH and HCl were from Nanjing Chemical Reagent Co. (Nanjing, China). Ultra-pure water was from the laboratory device.

### 2.2. Synthesis of Adsorbent Materials

**Figure 1:**
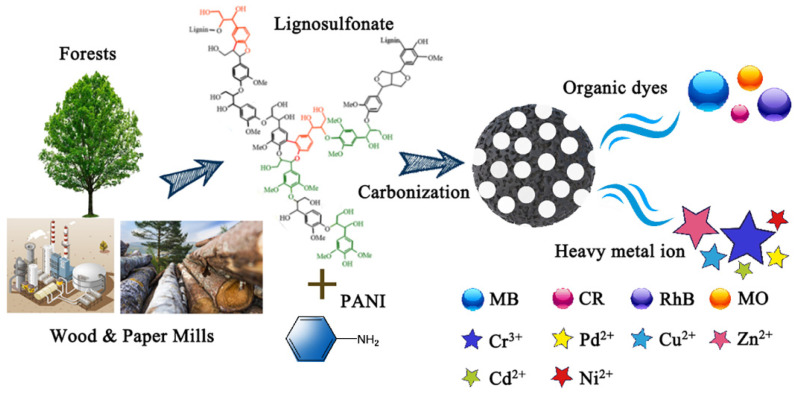
Flow chart of SNC preparation and adsorption application.

The carbon material used for adsorption was obtained via two-step calcination, and we prepared an oxygen/nitrogen/sulfur co-doped carbon material for the adsorption of dyes and heavy metal ions using polyaniline as the N source and lignosulfonate as the S source, and the specific steps and processes are shown in Figure 1.

Firstly, we prepared lignosulfonate/polyaniline composites in a previous stage [27], and then, the composite was pre-carbonized. The composites were vacuum-dried for 12 h, placed in a tube furnace under a nitrogen atmosphere at 400 °C for carbonization, and kept for 1.5 h. The carbonized product was ground to powder using mortar and pestle.

Next, carbon materials were activated with KOH. The KOH and carbon material mass ratio was a 1:1 mix, to which distilled water was added and stirred to a uniform solution and then dried to a solid. It was heated to 700 °C in a tube furnace at a 5 °C/min heating rate under inert gas and carbonized for 2 h. The carbonization of the product was washed with weakly acidic 1 M HCl and then rinsed several times with distilled water. The product of carbonization was pulverized to a powder using a mortar and pestle, after which it was dried until a constant mass was obtained. Finally, the O/N/S co-doped porous carbon material was obtained. When the carbonization temperatures were 700 °C, they were labeled SNC, and the undoped lignin was NC [28]. SNC was chosen as the adsorbent material, and NC was used as the control group.

### 2.3. Characterization

The surface morphology of SNC and SNC after adsorption of dyes and the elemental distribution of EDS were observed using a field emission scanning electron microscope (SEM, FEI Quanta 200, Hillsboro, OR, USA). X-ray photoelectron spectroscopy (XPS, AXIS Ultra DLD, Kratos, Manchester, UK) was used to investigate the surface chemical properties of SNC and SNC after the adsorption of dyes, including the relative content of elements as well as the type and relative content of chemical functional groups. The surface chemistry of SNC and SNC after dye adsorption was investigated, including the relative elemental content as well as the type and relative content of chemically functional groups. The specific surface area and pore size distribution of SNC were calculated by measuring the nitrogen adsorption–desorption isotherms using Brunner–Emmett–Taylor analysis (BET, QUADRASORB-EVO, Quantachrome Instruments, Boynton Beach, FL, USA). The physical structure of SNC is shown in the Supplementary Materials Figure S1 [28].

Adsorption methods are described in the Supplementary Materials. The adsorption processes and mechanisms are explained via kinetic, isothermal, and thermodynamic modeling fits and combinations [29,30].

The adsorbed amount of the dye and the rate of removal were calculated according to the following Equations (1) and (2).

(1) $$Q_{t}=(C_{0}-C_{t})V/m$$

(2) $$C\%=\frac{\left(C_{0}-C_{t}\right)}{C_{0}}\times 100.$$

Thermodynamic adsorption experiment:

A single variable method was adopted. A total of 0.1 g of methylene blue dye was dissolved in 1000 mL of distilled water to prepare a 100 mg/L stock solution, diluted into different initial concentrations (50, 100, 150, 200, 250, 300, 350, 400, 500, 600, 700, and 800 mg/L) of methylene blue solution. Weighed 10 mg of the materials NC700 and SNC700, respectively. Accurately weighed 10 mg of material NC700 and SNC700 were placed in 25 mL of MB solution, respectively, and the adsorption was carried out via oscillation in a thermostatic oscillator at a vibration rate of 150 rpm/min for 1 h. Afterward, the adsorption was carried out in the methylene blue solution with an initial concentration of 200 mg/L in a thermostatic oscillator at 85 °C, 70 °C, 55 °C, 40 °C, 25 °C, and 10 °C (i.e., 358, 343, 328, 313, 298, and 283 K) with a vibration speed of 150 rpm/min. The adsorption was carried out in a constant temperature oscillator at an oscillation speed of 150 rpm/min for 1 h. In all the above samples, the absorbance after adsorption was measured, and the corresponding adsorption concentration was calculated from the standard curve (Figure S4).

Kinetic adsorption experiment:

A total of 10 mg of the material was weighed and placed in 25 mL of MB solution with an initial concentration of 200 mg/L and placed in a thermostatic oscillator at 25 °C with a vibration speed of 150 rpm/min for adsorption for 10, 20, 40, 60, 80, 100, and 120 min, respectively. Then, the absorbance after adsorption was measured, and the corresponding adsorption concentration was calculated from the standard curve. The adsorption amount and removal rate of the dye were calculated according to the following Equations (1) and (2).

Rhodamine B, Congo red, and methyl orange have the same adsorption principle and similar steps. As shown in Figure S2, the maximum absorption wavelength of methylene blue is 664 nm, the UV absorption wavelength of Congo red is 488 nm, the maximum absorption peak of MO is about 466 nm, and the absorbance at the maximum wavelength of rhodamine B is 492 nm.

Desorption after SNC adsorption: After 2 h of adsorption, the solids were filtered to obtain a sample of the SNC adsorbed dye, and the SNC carbon material with adsorbed dye was treated with 25 mL of 0.1 mol/L HCl and 10% ethanol solution to wash away the dye stored in the SNC. The desorption rate (Des) of the dye was calculated as (Equation (3)):

(3) $$Des(\%)=\frac{Concentration\;of\;dye\;desorbed\;by\;0.1\;mol/L\;HCl}{Initial\;concentration\;of\;dye\;adsorbed\;on\;SNC}\times 100\%.$$

Adsorption of heavy metals: In the first stage, the samples were placed in a Pb^2+^ standard solution (which can be diluted to different concentrations according to the actual situation) for adsorption. Then, the Cr^3+^, Pb^2+^, Ni^2+^, Cu^2+^, Zn^2+^, and Cd^2+^ concentration in the supernatant was detected using an inductively coupled plasma mass spectrometer (ICP-MS, iCAP RQ, Thermo Scientific, Dreieich, Germany). To ensure accuracy, each experiment was conducted three times. The formula for the adsorption capacity Q_e_ (mg/g) of LS/PANI can be calculated with reference to that of the dye above.

## 3. Results and Discussion

### 3.1. Effect of Time on Adsorption Performance and Adsorption Kinetic Study

Kinetic studies help understand the optimum time when adsorption equilibrium is reached, which is important in analyzing the adsorption mechanism. The efficiency of an adsorbent depends not only on the adsorption capacity but also on the rate of uptake of pollutants from wastewater.

The kinetics of the two dyes were further investigated based on the data from the pseudo-first-order model and pseudo-second-order kinetic model, which are shown in Equations (4) and (5), respectively. Based on the pseudo-first-order kinetic model and the pseudo-second-order kinetic model, respectively, the experimental data were processed using a linear fitting method to analyze the adsorption process of the SNC material.

Pseudo-first-order kinetic model:

(4) $$\ln(q_{e}-q_{t})=\ln q_{e}-k_{1}t$$

where

q_e_—equilibrium adsorption amount, mg/g;
k_1_—adsorption rate constant, min^−1^;
q_t_—adsorption amount at time t, mg/g.

Pseudo-second-order kinetic model:

(5) $$\frac{t}{q_{t}}=\frac{1}{k_{2}q_{e}^{2}}+\frac{t}{q_{e}}$$

where

q_e_—equilibrium adsorption amount, mg/g;
k_2_—adsorption rate constant in this model, g/mg/min;
q_t_—adsorbed amount per unit mass of adsorbent at any adsorption time t, mg/g.

Particle diffusion equation:

(6) $$q_{t}=k_{d}t^{\frac{1}{2}}+C.$$

**Figure 2:**
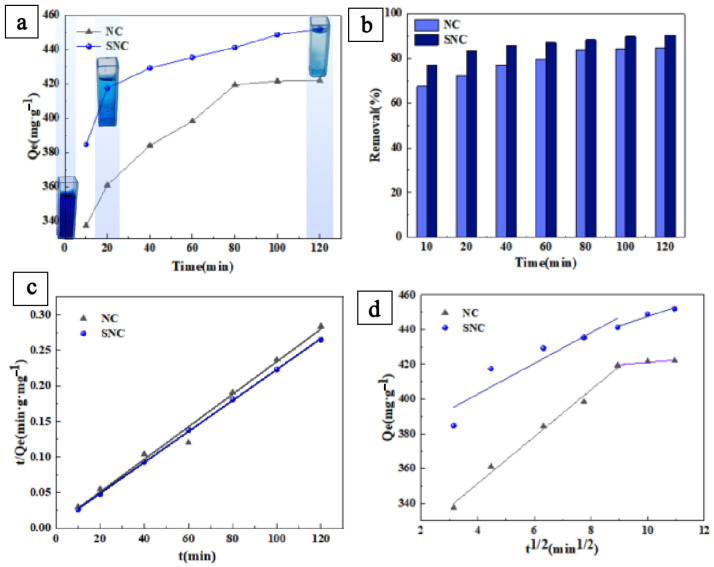
(**a**) Influence of time on adsorption capacity of MB (the inset is a digital photo of the SNC before and after adsorption); (**b**) effect of time on removal rate of MB; (**c**) linear fitting of NC and SNC in pseudo-second-order dynamics of MB; (**d**) linear fitting diagram of NC and SNC intra-particle diffusion models.

Adsorption time is an important parameter to study the adsorption kinetics. In a MB solution, with the increase in adsorption time, the removal rate and adsorption amount of MB increased gradually. The removal rate of the dye could reach 90.41%, whereas the removal rate of NC could only reach 84.42% (Figure 2a,b). Through the study of kinetic model parameters in Table 1, the pseudo-second-order kinetic model of SNC (R^2^ ≥ 0.97) fitted better than the pseudo-first-order kinetic model (R^2^ ≥ 0.82), and the actual adsorption amount did not differ much from the pseudo-second-order kinetic theoretical adsorption amount (453.42 mg/g), and the linear fitting to the pseudo-second-order kinetic data as shown in Figure 2c, the correlation coefficient was still above 0.99. This result suggests that this adsorption process is chemisorption. MB is a cationic molecule, and the adsorption on carbon is carried out through the processes of electrostatic interaction, hydrogen bond formation, electron donor-acceptor relationship, and π–π electron dispersive force between functional groups on the surface of carbon and MB molecules. In addition to the specific surface area and porosity of the carbon adsorbent, groups such as aromatic rings, -C=O, -C-O-C-, -OH, -NH_2_, -C=S, -C=N, -S=O, and so on, play an important role in enhancing the adsorption capacity of MB in water [31,32]. It is known that the synthesized SNC materials have most of the groups such as -C=O, -C=S, and -C=N, which can form chemical bonds with dye molecules.

As shown in Figure 2d, using the intra-granular diffusion model to analyze the possible diffusion mechanism of carbon materials, the results from the data parameters in Table 2 can show that the adsorption process is a segmented linear relationship, including two main stages: the first stage is for the membrane diffusion stage of the dye molecules, the curve rises faster, $K_{i1}$ are greater than $K_{i2}$, for physical adsorption. The second stage is progressive adsorption, where the adsorption rate slows down and finally reaches the adsorption equilibrium. Each stage did not pass through the origin, indicating that the intra-granular diffusion is not the only quick-control step, and the adsorption process may involve other complex reactions.

### 3.2. Effect of Concentration and Temperature on Adsorption Performance and Thermodynamic Study of Adsorption

Freundlich assumed that adsorption can occur through the formation of multilayers on the nonhomogeneous surface of the adsorbent. The equation of the Freundlich isothermal adsorption model (Equation (7)):

(7) $$\ln Q_{e}=\ln K_{F}+\frac{1}{n}\ln C_{e}$$

where

Q_e_—equilibrium adsorption amount, mg/g;
C_e_—equilibrium concentration, mg/L;
K_F_—adsorption equilibrium constant;
n—intensity factor.

Langmuir assumes that a monomolecular layer adsorption is formed on the surface of the adsorbent; furthermore, it also refers to the adsorption of only one molecule of adsorbent at an adsorption site, resulting in a decrease in intermolecular forces with increasing distance. This isotherm also assumes that the adsorbent surface is homogeneous with similar and potentially equivalent adsorption sites. Langmuir isothermal adsorption model equations (Equation (8)):

(8) $$\frac{C_{e}}{Q_{e}}=\frac{1}{K_{L}Q_{m}}+\frac{C_{e}}{Q_{m}}$$

where

K_L_—Langmuir constant, L/mg;
Q_m_ is the maximum adsorption capacity per unit mass of adsorbent, mg/g.

**Figure 3:**
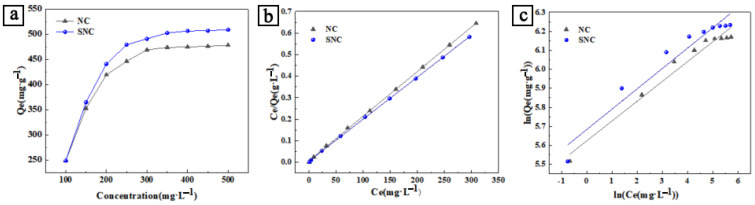
(**a**) Effect of initial concentration on adsorption capacity of MB; (**b**) linear fitting of Langmuir model in MB; and (**c**) linear fitting of Freundlich model in MB.

Figure 3a shows that the adsorption amount increased rapidly to the adsorption equilibrium with the increase in the initial concentration of dye. At an initial concentration of 500 mg/L, the adsorption equilibrium amount reached 478.30 mg/g and 509.00 mg/g for NC and SNC, respectively. The initial tortuosity indicated that as additional sites are filled in the adsorbate, it increases the challenge for a solute molecule to find a usable vacancy site [33]. However, this trend does not apply to all carbon adsorbents, where some of the high specific surface area carbons showed lower adsorption capacity, and the current adsorption was dominated by chemisorption.

Thermodynamically relevant parameters and models [29].

(9) $$K_{d}=\frac{{\mathrm{mq}}_{e}}{C_{e}V}$$

(10) $$\ln K_{d}=\frac{{\Delta S}^{0}}{R}-\frac{{\Delta H}^{0}}{\mathrm{RT}}$$

(11) $$\Delta G^{0}=\Delta H-T\Delta S$$

where

R—standard molar constant, 8.314 × 10^−3^ J/(mol·K);
ΔG^0^—Gibbs free energy, kJ/mol;
ΔS^0^—standard entropy change, kJ/mol;
ΔH^0^—standard enthalpy change, kJ/mol;
K_d_—partition coefficient;
m—mass of adsorbent, g;
V—volume of dye solution, L.

Methylene blue adsorption isotherm is a component of thermodynamic adsorption. It describes the equilibrium relationship between the amount of MB adsorbed on NC and SNC adsorbed at a certain temperature and the amount adsorbed in the solution. Figure 3b,c were further linearly fitted to validate the model’s reasonableness. When modeling the MB dye, the adsorption process is more in line with the Langmuir adsorption isothermal model, as can be seen from Table 3 correlation coefficients, where MB forms a homogeneous monolayer coverage on the surface of the carbon material, and there are no further interactions between adsorbent layers. In addition, Q_m_, which can also be calculated from Table 3, the value of dye adsorption using the Langmuir model was 671.78 mg/g, which is higher than that of NC, and the difference in the adsorption capacity may be attributed to the excellent pore size of SNC. The reason for the larger Q_m_ may be that the adsorbent SNC has many carboxyl, sulfonate, amine, and phenyl functional groups, which can easily form electrostatic interactions, boosting the MB uptake capacity. It is, of course, possible that at higher MB dye concentrations, multilayer adsorption of MB is possible via stacking of planar molecules. The *n* values of the Freundlich isotherms are in the range of 2.97–3.19, which indicates that the adsorption of MB is a favorable process [34].

**Figure 4:**
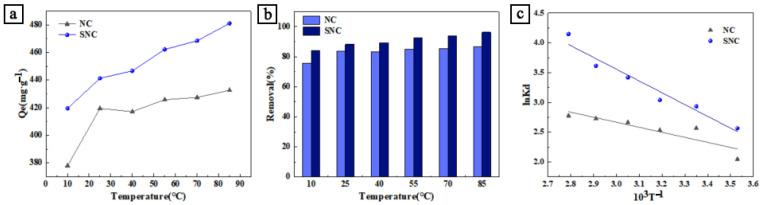
(**a**) Effect of temperature on adsorption capacity of MB; (**b**) effect of temperature on removal rate of MB; (**c**) and adsorption of MB InK_d_ and 1/T curve diagram.

From Figure 4a,b, it can be seen that the increasing temperature accelerated the adsorption rate of the carbon material and increased the adsorption amount, and the SNC adsorption amount reached 480.99 mg/g at 85 °C, and the removal rate also showed an increasing trend until 96.2%. According to Equations (9)–(11), the summarized information of the thermodynamic parameters, as shown in Table 4, can be obtained. Since $\Delta G^{0}$ < 0, $\Delta H^{0}$ > 0, and $\Delta S^{0}$ > 0 confirms that the adsorption of MB on the carbon-based adsorbent is a spontaneous and feasible adsorptive heat transfer process, and the reaction increases the disorder of the interface between the solid–liquid two-phase. The absolute values gradually increase, and the driving force for the adsorption of MB increases with increasing temperature. During adsorption, MB molecules are adsorbed from the solution onto the surface of the SNC. When MB molecules are adsorbed onto the surface of the adsorbent, the state of the system changes, and the number of states of the system associated with the dye molecules decreases since the adsorption sites on the surface of the SNC are limited. The adsorption process itself also leads to an increase in the disorder of the system, which results in an increase in entropy.

### 3.3. The Applicability of SNC to the Adsorption of Various Pollutants and Cyclic Performance

**Figure 5:**
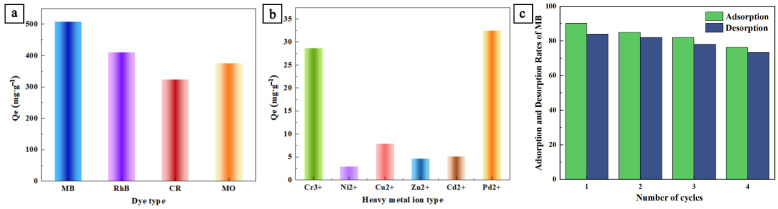
(**a**) Adsorption of different kinds of dyes by SNC; (**b**) adsorption of different kinds of heavy metal ions by SNC; (**c**) and cyclic performance of adsorption and desorption of SNC.

In addition, the adsorption capacity of SNC for RhB, CR, and MO at equilibrium was 410.2, 323.6, and 375.4 mg/g, respectively, at an initial concentration of 500 mg/L. The adsorption performances of SNC for various cationic and anionic dyestuffs were very good, but the cationic dyestuffs were significantly better than the anionic dyestuffs. None of the differences in the adsorption capacity were very large, so the molecular structure and size of the dyes were the main reasons that would play a role. We also tested the adsorption capacity of SNC for various metal ions and organic dyes (Figure 5a,b). The adsorption capacities of SNC for Cr^3+^, Pb^2+^, Ni^2+^, Cu^2+^, Zn^2+^, and Cd^2+^ were 28.7, 32.4, 2.9, 7.9, 4.7 and 5.1 mg/g, respectively. It can be seen from these results that the SNC has strong adsorption capacity for Pb^2+^ and Cr^3+^ and presumably also has good selectivity. The amino group of SNC is the critical base and can complex well with the boundary acid. Pb(II) has the lowest hydration energy and can bind stably with the amino group. It is also stated in the theory of HSBA that Cu^2+^, Pb^2+^, and Zn^2+^ are the critical acids, Cd^2+^ is the soft acid, and Cr^3+^ is a hard acid [35]. Therefore, SNC has better adsorption properties for Pb^2+^. The maximum values of adsorption per unit mass of lignin-based carbon material adsorbent reported in the recent literature are summarized in Table 5 [26,36,37,38,39,40,41,42,43,44,45,46,47,48,49,50]. As can be seen from Table 5, the values of dyes and heavy metals adsorbed in the present study are comparable to exceeded by other reported adsorbents of lignin-based carbon material. However, compared to MOF, porous polymers, and mesoporous silica, lignin/PANI-based carbon materials still have some shortcomings (e.g., in terms of specific surface area), but they have a wide range of functional groups that are beneficial for adsorption [51,52]. The recycling performance of adsorbents in water purification processes is an evaluation indicator for the development of cost-effective processes in today’s society. As shown in Figure 5c, we cycled the SNC adsorption/desorption four times and used 0.1 mol/L HCl and 10% ethanol as eluent [53], and the MB removal decreased from 90.2% to 76.4% after four cycles with good reusability. Therefore, SNC can be considered as an economical adsorbent.

### 3.4. Adsorption Mechanism and Physical Properties of MB and Pb^2+^ by SNC

**Figure 6:**
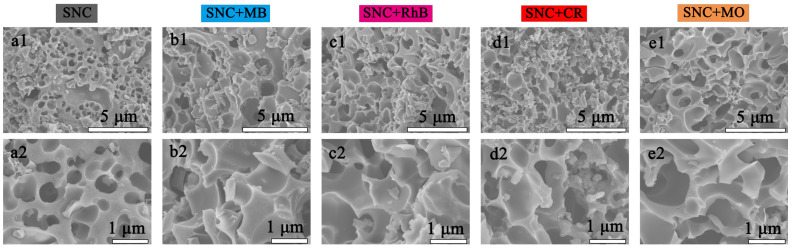
SEM images for SNC before and after adsorption of the dyes, (**a**) SNC; (**b**) SNC+MB; (**c**) SNC+RhB; (**d**) SNC+CR; and (**e**) SNC+MO.

**Figure 7:**
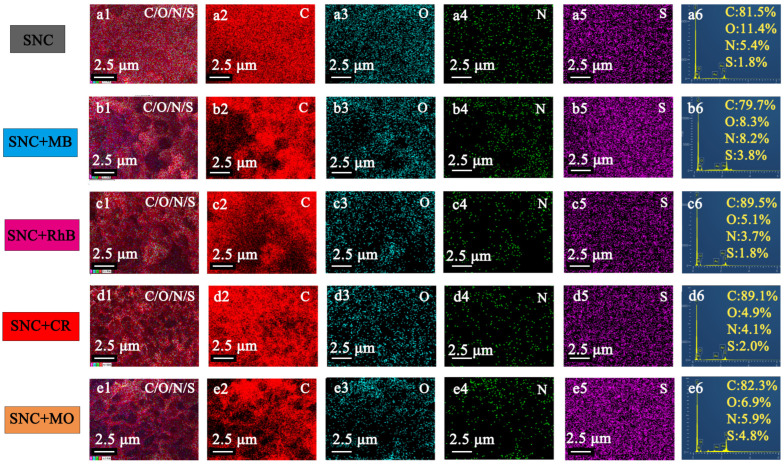
EDX analysis for SNC before and after adsorption of the dyes, (**a1–a6**) SNC; (**b1–b6**) SNC+MB; (**c1–c6**) SNC+RhB; (**d1–d6**) SNC+CR; and (**e1–e6**) SNC+MO.

As shown in Figure 6a–e, the SEM surface morphology shows that the pore distribution of SNC is uniform and the porosity is high, and after the adsorption of MB, RhB, MO, and CR dyes on SNC, the surface morphology is filled with small molecules, and the pores are filled with small molecules as well. The elemental composition (C, N, O, and S) in SNC, SNC+MB, SNC+RhB, SNC+CR, and SNC+MO was investigated using EDS spectroscopy, as shown in Figure 7a–e. The content of O decreased from 11.4% to 8.3%, 5.1%, 4.9%, and 6.9% after adsorption, which may be attributed to the fact that the carbon material has more oxygen-containing functional groups in the lignin during the calcination process, and after adsorption of the dye molecules aggregated on the surface, and the dye molecules were less oxygenated. The contents of C, O, N, and S in SNC+MB were 79.7%, 8.3%, 8.2%, and 3.8%, respectively. The uniform distribution of dye on the adsorbed surface can be seen from the EDX (Figure 7).

**Figure 8:**
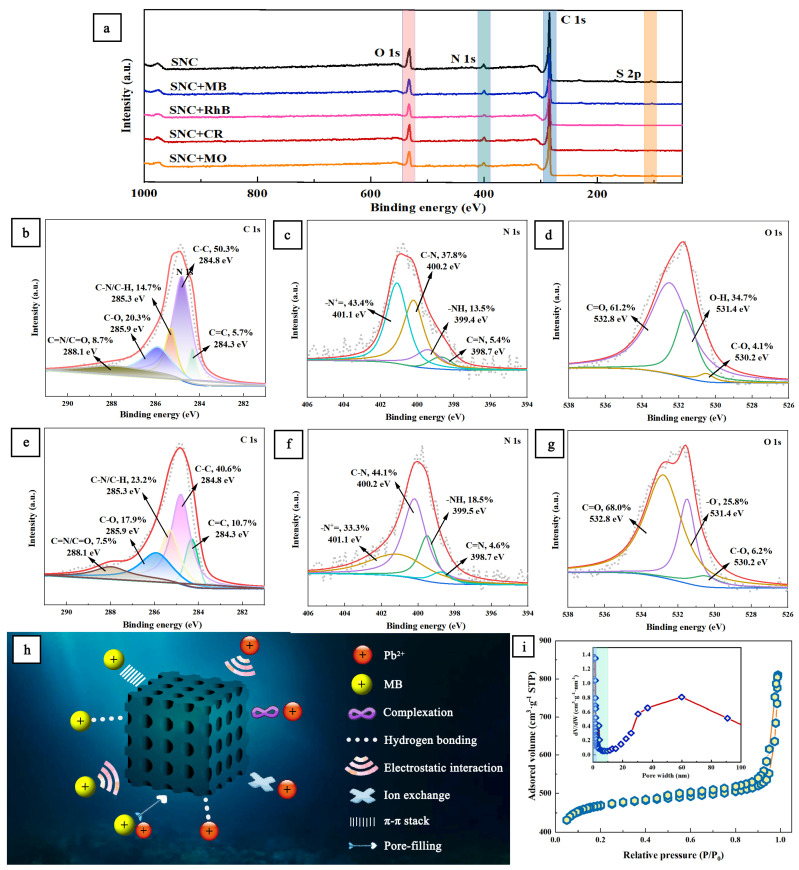
(**a**) XPS survey spectra of SNC and SNC+MB, SNC+RhB, SNC+CR, and SNC+MO; high-resolution XPS of C 1s (**b**), O 1s (**c**), and N 1s (**d**) for SNC; high-resolution XPS of C 1s (**e**), O 1s (**f**), and N 1s (**g**) for SNC+MB; (**h**) predicted adsorption mechanism of SNC on cationic dyes and heavy metal ions; and (**i**) the N_2_ adsorption–desorption isotherms, pore size distributions.

As shown in Figure 8a, the peaks of C, O, N, and S appeared in the XPS spectra of SNC, SNC+MB, SNC+RhB, SNC+MO, and SNC+CR, with the highest intensity of the oxygen peak in SNC, which is consistent with the EDS results. As shown in Figure 8b, there are five peaks in the C 1s XPS spectra of SNC: 8.7% in the C=N/C=O group at 288.1 eV, 20.3% in the C-O group at 285.9 eV, 14.7% in the C-N/C-H group at 285.3 eV, 50.3% in the C-C group at 284.8 eV, and 5.7% in the C=C group at 284.3 eV. As shown in Figure 8b, after the adsorption of MB, chemical reactions such as complexation and π–π conjugation occurred between some C atoms, resulting in significant changes in the peak area and binding energy of C 1s and a decrease in the C-C density. As shown in Figure 8c, there are four peaks in the XPS spectrum of N 1s of SNC: 43.4% of -N^+^= group at 401.1 eV, 37.8% of C-N group at 400.2 eV, 13.5% of -NH- group at 399.4 eV, and 5.4% of C=N group at 398.7 eV. As shown in Figure 8f, after the adsorption of MB, the -NH groups decreased, probably due to the hydrogen bonding between the -NH groups and MB. As shown in Figure 8d, the O 1s XPS spectrum of SNC has three peaks: 61.2% of C=O groups at 532.8 eV, 34.7% of O-H groups at 531.4 eV, and 4.1% of C-O groups at 530.2 eV. As shown in Figure 8g, after the adsorption of MB, the density of O-H groups decreases from 34.7% to 25.8%, but that of C=O groups increases significantly from 61.2% to 41%. 61.2% to 68.0%. The changes in the peak area and binding energy of -OH are due to the hydrogen bonding between -OH and -S, -NH, and N=N in MB [54].

Figure 8h shows the adsorption mechanism diagram of the presumed SNC adsorption of the dye MB and the heavy metal ion Pb^2+^ [36]. Due to the presence of lignosulfonates, the SNC has sulfonic acid groups and hydroxyl, amino, and carbonyl groups, and all of these functional groups are involved in the adsorption of Pb^2+^. Amino groups on the SNC will provide the active site for binding to metal ions. For example, Pb^2+^ can be adsorbed onto the SNC through N and O complexation with the amino, carboxyl, and hydroxyl groups. The carboxyl and hydroxyl groups can bind Pb^2+^ by electrostatic attraction and ion exchange [36]. Hydrogen bonding connections and π–π stacking also exist between MB and SNC. In addition, SNC has a high specific surface area (511 m^2^/g, Figure 8i), so there will be pore filling [28]. In summary, the mechanisms of Pb^2+^ adsorption via SNC include ion exchange, complexation, and electrostatic interaction, and the mechanisms of MB adsorption include π–π conjugation, pore filling, hydrogen bonding, and electrostatic attraction.

## 4. Conclusions

In this study, oxygen–nitrogen–sulfur co-doped porous carbon materials prepared from lignin were used as effective dye adsorbents to study the adsorption properties of MB dye. The optimum adsorption conditions for removing MB dye were as follows: adsorbent dosage of 10 mg, initial dye concentration of 500 mg/L, adsorption time of 120 min, and temperature of 85 °C. The adsorption of MB dye by nitrogen and sulfur co-doped porous carbon materials conformed to the Langmuir adsorption model and pseudo-second-order adsorption kinetic model and belonged to the monomolecular layer adsorption dominated via chemical adsorption. Moreover, we tested the adsorption performance of SNC on different dyes and heavy metal ions and found that SNC had the best adsorption effect on cationic dyes (MB, RhB), and the highest adsorption on Cr^3+^, Pb^2+^ ions, which were 509.0 mg/g, 410.2 mg/g, 28.7 mg/g, and 32.4 mg/g, respectively. In addition, SNC also has good recycling performance and can be used as an effective adsorbent for removing organic dyes and heavy metal ions from water.

## Figures and Tables

**Table 1:** NC and SNC kinetic model fitting parameters.

Samples	Pseudo-First-Order Adsorption Kinetic Model			Pseudo-Second-Order Adsorption Kinetic Model		
	$Q_{1}$ (mg·g^−1^)	$k_{1}$ (min^−1^)	R^2^	$Q_{2}$ (mg·g^−1^)	$k_{2}$ (g·mg^−1^min^−1^)	R^2^
NC	405.65	0.16	0.67	426.69	7.79 × 10^−3^	0.91
SNC	439.31	0.20	0.82	453.42	1.22 × 10^−3^	0.97

**Table 2:** Parameters of NC and SNC intra-particle diffusion models.

Samples	$K_{i1}$ (mg·g^−1^·min^−0.5^)	$C_{1}$ (mg·g^−1^)	$R_{1}^{2}$	$K_{i2}$ (mg·g^−1^·min^−0.5^)	$C_{2}$ (mg·g^−1^)	$R_{1}^{2}$
NC	13.51	297.29	0.99	1.36	407.53	0.89
SNC	8.85	367.38	0.86	5.40	393.58	0.96

**Table 3:** Isotherm parameters of NC and SNC adsorption of MB.

Samples	Langmuir Adsorption Isotherm Model			Freundlich Adsorption Isotherm Model		
	$K_{L}$ (g·L^−1^)	$Q_{m}$ (mg·g^−1^)	R^2^	$K_{F}$$\;(mg^{1-1/n}\cdot L^{-1/n}$)	n	R^2^
NC	8.85 × 10^−3^	615.72	0.91	73.02	3.19	0.81
SNC	7.92 × 10^−3^	671.78	0.91	67.49	2.97	0.82

**Table 4:** Thermodynamic parameters of NC and SNC adsorption of MB.

Samples	$\Delta G^{0}/(\mathrm{kJ}\cdot {\mathrm{mol}}^{-1})$						$\Delta H^{0}/(\mathrm{kJ}\cdot {\mathrm{mol}}^{-1})$	$\Delta S^{0}(J\cdot {mol}^{-1}\cdot K^{-1})$
	283 K	298 K	313 K	328 K	343 K	358 K		
NC	−5.22	−5.87	−6.51	−7.16	−7.81	−8.46	7.02	43.24
SNC	−5.88	−7.07	−8.25	−9.44	−10.62	−11.80	16.45	78.92

**Table 5:** Partial research progress on adsorption of dyes and heavy metal ions by lignin-based carbon material adsorbents.

Lignin-Based Carbon Materials	Adsorption of Dye/Heavy Metal Ions	Adsorption Capacity (mg/g)	Ref.
Cork activated carbon	RhB	1734.6	[36]
	Pb(Ⅱ)	231.5	
Lignin nanoparticle-g-polyacrylic acid adsorbent	Safranin-O	138.9	[37]
Lignin-based few-layered graphene-encapsulated iron nanoparticles	As(III)	214.7	[38]
FeS@Lignin-derived carbon	Tellurium (IV)	148.4	[39]
Lignin-derived mordenite templated carbon	MO	225.0	[40]
Cu/N-doped lignin	As(V)	253.5	[26]
Lignin-derived sulfonated porous carbon	MB	234.2	[41]
Carbon-Fe_3_C/lignin composites	Cr(VI)	164.0	[42]
Black liquor lignin	MB	92.5	[43]
Bio-based lignin/chitosan adsorbent	CR	173.0	[44]
Magnetic mesoporous sodium citrate-modified lignin	Ca(II)	339.4	[45]
	MB	281.4	
Lignin-based magnetic nanoparticle adsorbent	MB	234.3	[46]
Lignin-derived magnetic activated carbons	MB	220.2	[47]
Carbon nanofibers from a blend of lignin	Pb(II)	147.8	[48]
Lignin-based porous carbon with layered graphene-like structure	Pb(II)	250.5	[49]
Activated carbon prepared from natural lignin	MB	147.0	[50]
Sodium lignosulfonate/polyaniline composite as the precursor, the activated high-temperature pyrolysis process is used to prepare porous carbon materials with oxygen, sulfur and nitrogen content	MB	509.0	This work
	RhB	410.2	
	CR	323.6	
	MO	375.4	
	Cr(III)	28.7	
	Ni(II)	2.9	
	Cu(II)	7.9	
	Zn(II)	4.7	
	Cd(II)	5.1	
	Pb(II)	32.4	

## Data Availability

Data are contained within the article.

## Supplementary Materials

The following supporting information can be downloaded at https://www.mdpi.com/article/10.3390/polym15234515/s1, Figure S1: (a) Raman spectra of SNC700; (b) XRD patterns of NC and SNC; Figure S2: Absorbance curves of MB, CR, MO, RhB; Figure S3: Absorbance curves of SNC before and after adsorption of four dyes; Figure S4: MB standard curve; Table S1: Organic dyes with different charge types and sizes; Table S2: Information on the molecular structure of different heavy metal ions.
