# Supplementary material for: Polyaniline as a Nitrogen Source and Lignosulfonate as a Sulphur Source for the Preparation of the Porous Carbon Adsorption of Dyes and Heavy Metal Ions
Source: Polymers (Basel). 2023 Nov 24;15(23):4515. doi: 10.3390/polym15234515 (PMC10708433; doi:10.3390/polym15234515)
Supplement: Supplementary file 1 [file polymers-15-04515-s001.zip › polymers-2661455-supplementary.pdf]

Supporting Information

Polyaniline as a Nitrogen Source and Lignosulfonate as a Sulphur Source for the Preparation of the Porous Carbon Adsorption of Dyes and Heavy Metal Ions

Wenjuan Wu [1,2,*] Penghui Li [1,2], Wanting Su [2], Zifei Yan [2], Xinyan Wang [2], Siyu Xu [1], Yumeng Wei [2] and Caiwen Wu [1,2]

[1] Jiangsu Co-Innovation Center of Efficient Processing and Utilization of Forest Resources, Nanjing Forestry University, Nanjing, 210037, P.R.China.

[2] College of Light Industry and Food Engineering, Nanjing Forestry University, Nanjing, 210037, P.R.China.

1 Material Characterization

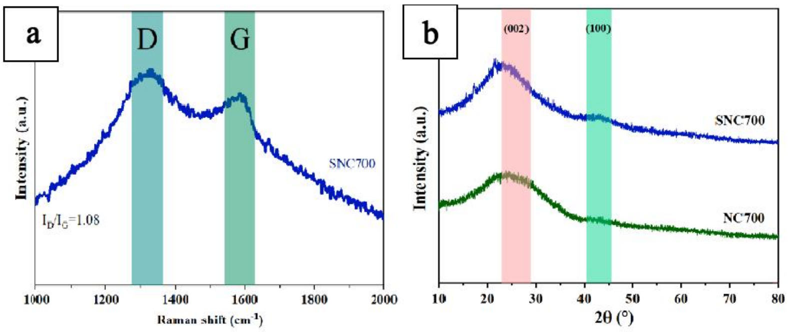

Fig. S1 (a) Raman spectra of SNC700; (b) XRD patterns of NC and SNC [28].

2 Adsorption measurement

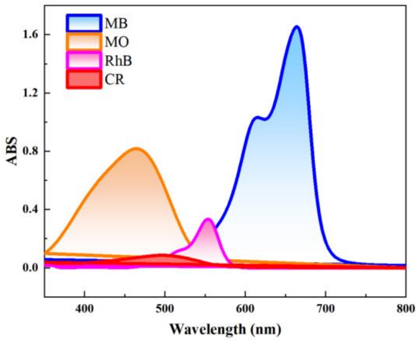

Fig. S2 Absorbance curves of MB, CR, MO, RhB

Evaluation of the adsorption selectivity of SNC for four dyes: Firstly, 0.1 mg of methylene blue, 0.1 mg of rhodamine B, 0.1 mg of Congo red and 0.1mg of methyl orange dyes were dissolved in

10 ml of water, and the UV spectra were measured at 200-800 nm. Afterwards, the four solutions were mixed together, and the UV spectra were measured at 200-800 nm.

Then, the four solutions were mixed and 0.5 mg of SNC was weighed and put into the mixed solution, waited for half an hour and measured 200-800 nm.

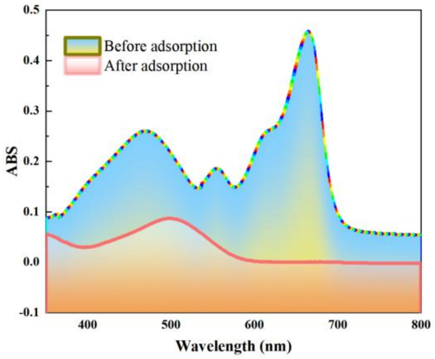

Fig. S3 Absorbance curves of SNC before and after adsorption of four dyes

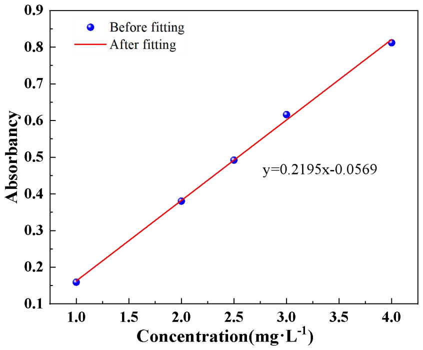

Fig. S4 MB standard curve

Table S1 Organic dyes with different charge types and sizes

Dye	Formula	Charge type	Size (nm × nm × nm)
Methylene blue	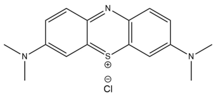	Cationic	0.40 × 0.79 × 1.63
Rhodamine B	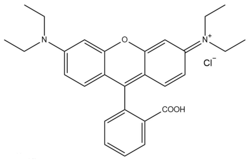	Cationic	0.68 × 1.18 × 1.57
Methyl orange	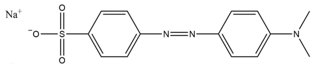	Anionic	0.53 × 0.73 × 1.74
Congo red	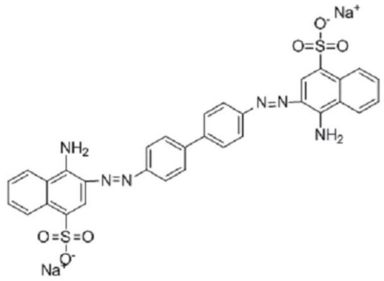	Anionic	0.39 × 0.86 × 2.61

Table S2 Information on the molecular structure of different heavy metal ions

Heavy metal ion	The atomic weight of the corresponding element atomic weight
Cr^{3+}	52.00
Pd^{2+}	207.2
Cu^{2+}	63.55
Zn^{2+}	65.38
Cd^{2+}	112.41
Ni^{2+}	58.69
